# 882. Gender Disparities in Faculty Rank and Mentorship opportunities Within a Sample of US Academic Infectious Disease Divisions

**DOI:** 10.1093/ofid/ofad500.927

**Published:** 2023-11-27

**Authors:** Benjamin Hollenberg, Kelly Cawcutt, Jasmine R Marcelin, Andrea J Zimmer, Nicolas W Cortes-Penfield

**Affiliations:** Washington State University, Pullman, Washington; University of Nebraska Medical Center, Omaha, Nebraska; University of Nebraska Medical Center, Omaha, Nebraska; University of, Omaha, Nebraska; University of Nebraska Medical Center, Omaha, Nebraska

## Abstract

**Background:**

Gender inequities in promotion within academic medicine, including academic infectious diseases (ID), are well-documented. Effective mentorship is a key component of career advancement and data show that same-gender mentorship can be valuable for women junior faculty learning to navigate academia. We compared the gender, bibliometrics, and mentorship opportunity distributions of ID faculty at each academic rank (instructor vs assistant, associate, or full professor) across several US medical centers.

**Methods:**

We randomly selected five academic medical centers from the US World News & Reports top 100 medical school rankings that had ID division websites including a faculty listing with ranks and years since graduation. We recorded each physician faculty’s rank and gender based on the written biography, using a validated tool (genderize.io) to determine gender if not given, and collected each faculty’s traditional bibliometric data via Google Scholar.

**Results:**

We included 221 faculty (107 women) from five ID divisions (mean 44 faculty). Women accounted for 5/12 (42%) of instructors, 62/109 (57%) of assistant professors, 19/44 (43%) of associate professors, and 21/56 (38%) of full professors. Junior faculty women (instructor or assistant) had a median 0.6 potential same-gender senior mentors per mentee within their divisions versus 1.1 for men; associate professor women had a median 1.0 potential same-gender senior mentor per mentee versus 1.7 for men. Measures of academic productivity, such as annual publications, citations, and h-index growth since graduation from fellowship were similar between men and women.

**Conclusion:**

Our findings support published studies demonstrating lower proportions of women in successively senior positions within academic ID divisions despite similar scholarly activity and suggest that fewer within-institution opportunities for senior mentorship might be a contributing factor.

**Disclosures:**

**Andrea J. Zimmer, MD**, Allovir: Grant/Research Support

